# Comparison between short- and long-acting erythropoiesis-stimulating agents in hemodialysis patients: target hemoglobin, variability, and outcome

**DOI:** 10.1007/s11255-013-0640-7

**Published:** 2014-01-22

**Authors:** Bassam Bernieh, Samra Abouchacra, Yousef Boobes, Mohammad R. Al Hakim, Nico Nagelkerke, Ahmad Chaaban, Mohamad Ahmed, Qutaiba Hussain, Hanan El Jack, Faiz Abayechi, Imran Khan, Nicole Gebran

**Affiliations:** 1 Nephrology Department, Tawam Hospital in Affiliation with Johns Hopkins Medicine, P.O. Box 15258, Al Ain, United Arab Emirates; 2 Community Medicine, College of Medicine and Health Sciences, United Arab Emirates University, Al Ain, United Arab Emirates; 3 Pharmacy Department, Tawam Hospital in Affiliation with Johns Hopkins Medicine, Al Ain, United Arab Emirates

**Keywords:** Short, Long-acting ESAs, Target hemoglobin, Hemoglobin variability, Hemodialysis

## Abstract

**Purpose:**

Maintaining target hemoglobin (Hb) with minimal variability is a challenge in hemodialysis (HD) patients. The aim of this study is to compare the long- and short-acting erythropoietin-stimulating agents such as Aranesp and Eprex in achieving these targets.

**Methods:**

Randomized, prospective, open-labeled study of 24 weeks includes stable patients on HD >3 months, age >18 years, and on Eprex for >3 months. Patients were randomized into two groups: A-(Aranesp group):HD patients on Eprex Q TIW or BIW were converted to Aranesp Q weekly, by using the conversion factor of 200:1 and those on Eprex Q weekly to Aranesp Q 2 weeks; B-(Eprex group):patients continued on Eprex treatment. Hemoglobin target was set at (105–125 g/l). Primary end points were percentage of patients achieving target Hb, hemoglobin variability, and number of dose changes in each group.

**Results:**

This study consisted of 139 HD patients: 72 in the Aranesp and 67 in the Eprex—mean (SD) age 54 (16.2) years, 77 (55 %) males. About 46 % were diabetic. Target Hb achieved in 64.8 % of the Aranesp and 59.7 % in the Eprex (*p* = 0.006). Hb variability was less frequent in the Aranesp group (*p* = 0.2). Mean number of dose changes was 1.3 (0.87) in the Aranesp and 1.9 (1.2) in the Eprex (*p* < 0.001). There was 1 vascular access thrombosis in the Aranesp and 8 in the Eprex (*p* < 0.001). There was no difference in hospitalization and death number between the 2 groups.

**Conclusions:**

Aranesp Q weekly or every 2 weeks is more efficient in achieving target Hb, with less dose changes and minor vascular access complications.

## Introduction

The hormone erythropoietin is produced in the kidneys and stimulates the bone marrow production of red blood cells. Diseased kidneys do not release sufficient amounts of erythropoietin hormone; anemia can result and it is universal in end-stage renal disease (ESRD). Anemia secondary to chronic kidney disease (CKD) is associated with increased hospitalization and decreased survival [1, 2], increased burden of cardiovascular disease [3, 4], and reduced quality of life [5, 6]. With the growing prevalence of CKD [7, 8], new approaches are required to improve the efficiency of anemia management without increasing the workload of health care staff. Erythropoiesis-stimulating agents (ESAs) have been available for almost two decades and remain the central strategy for the treatment for anemia in patients with CKD. The use of ESAs in the management of renal anemia has been shown to improve survival, reduce cardiovascular morbidity, and enhance quality of life [9, 10]. Recombinant human erythropoietin (RHuEPO) such as Epo alfa has proven efficacy in treating anemia in CKD patients [10, 11]. Because of its relatively short half-lives [~24 h when administered via the subcutaneous (SC) route] [12], this agent is usually administered two or three times weekly. However, multiple weekly injections are inconvenient for patients and health care providers, particularly in the setting of chronic disease. Darbepoetin alfa (Darbe alfa) is a longer-acting ESA that contains two additional N-linked sialic acid-containing carbohydrate chains compared with endogenous erythropoietin and the standard rHuEPOs molecules [13–15], its serum half-life is markedly longer than that of the rHuEPO, and it also has greater erythropoietic activity [12, 13, 16, 17]. Some studies have shown that the mean terminal half-life of Darbe alfa is approximately 73 h following SC administration in peritoneal dialysis (PD) patients and patients with CKD not yet receiving dialysis (i.e., patients with CKD stage 3 or 4) [17–19]. Thus, Darbe alfa may be administered less frequently than standard rHuEPO (Epo) and still maintains adequate serum concentrations to stimulate erythropoiesis with target Hb levels [16, 20]. Extended administration intervals represent an opportunity to simplify treatment and improve the efficiency of anemia management.

Direct comparison between short- and long-acting ESAs in maintaining target Hb and stability is lacking. The aim of the current study is to compare in hemodialysis patients, between the long-acting ESA (Aranesp) and the short-acting ESA (Eprex), in achieving target Hb and stability, number of dose adjustment, converting factor, outcome, and the cost.

## Patients and methods

### Study population

Hemodialysis patients on regular dialysis program at a tertiary hospital. Inclusion criteria were as follows: stable patients, on regular hemodialysis for at least 3 months, age ≥18 years, and on Eprex treatment for more than 3 months. The exclusion criteria were as follows: the presence of acute illness, chronic blood loss, hemoglobinopathy, or malignancy.

The study was conducted in accordance with the Declaration of Helsinki and was approved by Al Ain Medical District Research Ethics Committee (AAMDREC). All patients gave written informed consent before participation.

### Study design

This was a conversion, comparative, prospective, randomized, and open-labeled study [All hemodialysis patients dialyzed in center and fulfilled the study inclusion criteria at the beginning of the study (November 2010) were randomized and included].

The patients were randomized into 2 groups: Group A: long-acting ESA (Aranesp) and Group B: short-acting ESA (Eprex). Group A patients were converted from Eprex TIW or BIW to once weekly of Aranesp and those on QW Eprex to Q every 2 weeks of Aranesp. The recommended dose conversion ratio on the European label (1 μg darbepoetin alfa for 200 IU Epotein alfa) was used, while Group B patients would be continued on the same Eprex regimen. (During the study, there was freedom to adjust the doses in both groups).

Efficacy was evaluated by target Hb that should be 110–120 g/l with a range of 105–125 g/l.

The primary end points of the study were evaluated by the percentage of patients reaching the target Hb in each group and the Hb stability in each group (Hb level, number of interventions to achieve this).

The secondary end points were as follows: the treatment cost in each group calculated as units/kg/week and mcg/kg/week, factor of conversion from Eprex to Aranesp group during the study (started at 200/1), number of hospitalizations, deaths, and vascular access thrombosis during the study in each group. There were 3 months of titration and 3 months of evaluation. Iron stores were maintained in both groups to achieve Ferritin level ≥200 μg/l and transferrin saturation ≥20 % (“Appendix”). Blood chemistry, bone profile, and iron profile were done on monthly basis, complete blood count (CBC) once every 2 weeks (Q2 W), and intact PTH (iPTH) every 3 months. Dialysis adequacy was measured as online (KT/V) and urea reduction ratio (URR).

### Statistical analysis

To find a difference in mean Hb concentration C between the two groups of 0.5 times the within-group standard deviation, with a power of 80 % and a significance level of 5 %, a total of 63 patients per group is needed. One hundred forty patients (who were fulfilling the study inclusion criteria) were recruited.

### Primary end points

1. Percentage of patients reaching the target Hb in each group, there was 3 months of titration and 3 months of evaluation. “Target Hb: was 105–125 g/l”.
2. The Hb stability (variability) was assessed by
  - Summing the square of the difference between Hb values and the previous Hb values (i.e., those 2 weeks earlier).
  - Closeness (C) to target was assessed by summing the square of the difference between observed Hb values and 115.
  - Time on target in each group.
3. Number of dose changes in each group during study duration.

### Secondary end points

1. Cost calculated as units/kg/week and mcg/kg/week during the study.
2. Factor of conversion from Eprex to Darbe group during the study, we started at 200/1.
3. Vascular access thrombosis in each group during the study period.
4. Number of hospitalizations and cause of hospitalization, in each group during the study period.
5. Number of deaths and cause of death, in each group during the study period.

Standard statistical tests such as independent sample *t* tests for comparing continuous outcome measures between two trial arms were used.

## Results

**Fig. 1:**
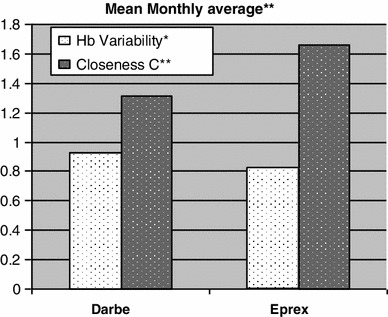
Mean monthly average of Hb variability and closeness C.*As a measure of Hb variability by using both sum and mean of the square of the difference between Hb values and the previous Hb values (i.e., those 2 weeks earlier) *p* = 0.08. **As a measure of closeness C to target by using the square of the difference between observed Hb values and 11.5, using nonparametric test (Mann–Whitney *U*) *p* = 0.09

**Fig. 2:**
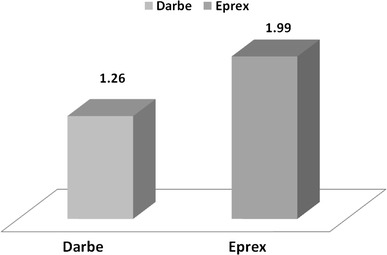
Number of dose changes during the evaluation phase (*p* < 0.01). Mann–Whitney test for general association of the ESA dose changing during evaluation phase between the two arms showed: *p* < 0.01

**Fig. 3:**
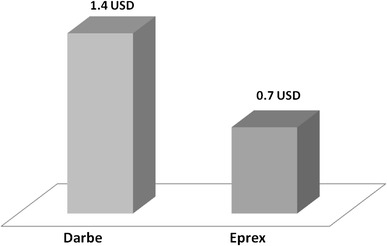
Average weekly cost/kg (*p* < 0.001). Mann–Whitney test for general association of the ESA cost between the two arms showed: *p* < 0.001

The data of 139 hemodialysis patients were analyzed, 72 (52 %) were in Aranesp group and 67 (48 %) were in Eprex group. The mean age was 54.4 (16.1) years, and there were 77 (55.4 %) males. Diabetes mellitus was the major cause of ESRD and encountered in 64 (45.7 %) of the patients. The majority of patients 121 (86.5 %) had no viral hepatitis while 15 patients (10.7 %) were HCV positive and 4 (2.8 %) patients were HBV positive. The vascular accesses AVF, TCC, and AVG were 97 (69.3 %), 23 (16.4 %), and 20 (14.3 %) in study cohort, respectively. The mean hemodialysis vintage was 57.7 (51.2) months. Table 1 shows the demographic data of both groups. Table 2 illustrates the baseline and, during the study, different biological parameters in both groups. The target hemoglobin (105–125 g/l) was achieved in 64.8 and 59.7 % in the Aranesp and Eprex groups, respectively, with significance difference between the two groups (*p* = 0.006); the details of the percentage of different Hb levels are shown in Table 3. Hb variability is a measure of both sum and mean of the square of the difference between Hb values and the previous Hb values (i.e., those 2 weeks earlier); closeness (C) which is the average of variability at each month of follow-up among study groups was calculated in each group, as shown in Fig. 1. Time on target was calculated as the average duration (in weeks) patients maintained on target Hb (10.5–12.5 g/dl); in the Darbe and Eprex groups, within the 6-month study duration, it was 16.8 and 15.3, respectively (*p* = 0.1). There was significant difference in the number of dose changes during the evaluation phase between the two groups, and it was 1.3 (0.87) in Darbe group and 2 (1.2) (*p* < 0.01) in the Eprex group, Fig. 2. The average weekly coast/kg was 1.4 USD in Darbe group and 0.7 USD in the Epo group, Fig. 3. Repeated-measure ANOVA for significant difference of the Darbe conversion factor throughout the study visits showed *p* < 0.001. Repeated-measure ANOVA for significant difference of the Eprex dose throughout the visits showed *p* = 0.606. There was 1 (1.4 %) vascular access thrombosis in the Darbe group and 8 (12 %) in the Eprex group (3 AVF, 3AVG, and 2 TC) (*p* < 0.001). There were 25 (35 %) and 29 (43 %) hospitalizations in the Darbe and Eprex groups, respectively (*p* = 0.56), and there were 2 deaths in each group during the study period (*p* = 0.99).

**Table 1:** Demographics data of both groups

	Aranesp		Eprex		Total	*p* value
Study population	72	52 %	67	48 %	139	–
Male	31	43 %	46	69 %	77 (55 %)	0.002
Female	41	57 %	21	31 %	62 (45 %)	
Age (years)	56.2	±17.56	52.5	±14.68		0.11
Nationality						
UAE	35	48.6 %	26	39 %	61 (44 %)	0.24
Non-UAE	37	51.4 %	41	61 %	78 (56 %)	
Etiology						
DM	34	47 %	30	44 %	64 (46 %)	0.40
Unknown	22	31 %	20	29 %	42 (30 %)	
Other	16	22 %	17	27 %	33 (24 %)	
Duration of hemodialysis (months)	56.5 (60)		50 (42)			0.98
Hepatitis status						
None	60	83 %	60	90 %	120 (86 %)	0.46
HCV +ve	10	14 %	5	7 %	15 (11 %)	
HBV +ve	2	3 %	2	3 %	4 (3 %)	
Vascular access^a^						
AVF	51	70 %	46	69 %	97 (70 %)	0.85
TC	12	17 %	10	15 %	22 (16 %)	
AVG	9	13 %	11	16 %	20 (14 %)	
Baseline dry weight (kg)	69 (18.3)		69 (17.5)			0.49

^a^ *AVF* arteriovenous fistula, *TC* tunneled catheter, *AVG* arteriovenous graft

**Table 2:** Biological parameters in both groups

Laboratory	Baseline					Mean value during study period				
	Aranesp		Eprex		*p* value	Aranesp		Eprex		*p* value
Ferritin	459.09	±277.69	464.16	±258.45	0.916	520.59	±255.85	528.39	±254.37	0.857
Transferrin saturation	30.31	±11.62	32.47	±16.6	0.393	33.30	±9.23	32.26	±9.22	0.505
Parathyroid hormone	50.5	±38.4	43.87	±43.5	0.881	52.93	±53.74	42.20	±33.31	0.179
Alkaline phosphatase	154.35	±182.63	168.23	±207.02	0.680	140.08	±113.68	150.84	±157.64	0.642
Albumin	33.69	±3.54	34.16	±3.53	0.433	33.82	±3.22	34.10	±3.24	0.608
Calcium (Ca)	2.26	±0.15	2.23	±0.15	0.136	2.30	±0.11	2.26	±0.14	0.055
Phosphate (PO4)	1.48	±0.41	1.5	±0.54	0.696	1.54	±0.36	1.57	±0.39	0.641
Sodium (N)	134.75	±3.07	135.09	±3.27	0.528	135.88	±2.22	135.80	±2.11	0.826
Potassium (K)	5.07	±0.64	5.09	±0.79	0.852	5.13	±0.48	5.15	±0.56	0.815
URR^a^	74.08 %	±11.94	74.86 %	±8.83	0.666	76.21 %	±5.85	75.56 %	±6.35	0.530
KT/V^b^	1.29	±0.21	1.33	±0.20	0.355	1.32	±0.19	1.32	±0.17	0.982

^a^ *URR* urea reduction ratio, a calculation measuring the dialysis efficiency (should be >66 %)

^b^ *KT/V* calculation, measuring the dialysis efficiency (should be >1.3)

**Table 3:** Target Hb in each group

Total Hb measures in 6 visits and mid-months	Darbe		Eprex		*p* value
Hb level					
Low (<10.5 g/dl)	91	11.3 %	128	16.8 %	0.006
Target (10.5–12.5 g/dl)	522	64.8 %	455	59.7 %	
High (>12.5 g/dl)	193	23.9 %	179	23.5 %	
Total (1,568 measures)	806	100 %	762	100 %

## Discussion

The National Kidney Foundation Kidney Disease Outcome Quality Initiative (KDOQI) recommends targeting Hb between 11.0 and 12.0 gm/dl, but evidence suggests that only 30 % of patients fall within this range at any point in time [10]. Despite improvements, large observational studies such as the Dialysis Outcomes and Practice Patterns Study (DOPPS) indicate that anemia remains prevalent in patients receiving dialysis; therefore, there is a need to increase the proportions of patients achieving guideline Hb targets [21]. Selection of the Hb target based on the patient’s disease state, comorbidities, and other characteristics has been an essential part of a treatment strategy [22]. However, the risks associated with high Hb targets in recent studies [23, 24] prompted updates to the guidelines to recommend a narrower Hb target: 110–120 g/l and not exceeding 130 g/l for most patients and 100–120 g/l for patients with type 2 diabetes mellitus (T2DM) avoiding levels above 120 g/l, particularly for those at risk of stroke [25]. In the current study, we have chosen the target of 110–120 g/l, with a margin of ±0.5 g/l, in line with the current recommendations [24]. The results of this study showed that both ESAs maintained the target Hb in almost 60 % of the patients, compared to the 40 % reported in large dialysis US patients [26]. However, the results illustrate the impact of the type of ESA used in maintaining target Hb; there was a significant difference between the percentages of patient achieving target Hb in the Darbe group compared to those in Epo group, and this difference was not demonstrated before. Hemoglobin variability assessed by different statistical methods showed a tendency of better Hb stability in the Darbe group compared to the Epo group, without reaching statistically significant difference between the two groups. Fluctuations in Hb levels result in frequent under- and overshooting of targets [27, 28]. Evidence suggests that hemoglobin variability does not only complicate maintenance of Hb within the target range, but it is also independently associated with mortality [29, 30]. On the other hand, stability of Hb in the target level (110–120 g/l) is associated with lower 1-year mortality risk in hemodialysis patients [29]. The number of intervention for dose changes of the long-acting Darbe group was significantly less compared to the Epo group. Data show that longer dosing intervals may lead to less variability in hemoglobin levels over time by producing fewer peaks and troughs and thereby requiring fewer dosage adjustments [31]. The recommended dose conversion ratio on the European label (1 μg darbepoetin alfa for 200 IU Epotein alfa) was used in this study; there was a steady increase in the conversion factor, with subsequent decrease in the Darbe dose, during the study period from 200:1 at baseline to 350:1 at 6 months, with an average of 268:1 at the end of the study, with significant difference from the baseline and the end (*p* < 0.001). This conversion ratio is higher than the average ratio reported in the meta-analysis of 21 studies, with 16,378 patients of 217:1 and that reported by the Canadian study of 169:1 [32]; this difference in the conversion factor could be explained by the different design of the studies (conversion vs. straight), geographic area, quality of dialysis, and center-related anemia management practice [33]. On the other hand, the dose of Epo remained stable during the study (*p* NS). The cost of Darbe was double that of Epo (1.4 vs. 0.7 USD/kg/week); however, several factors should be taken into consideration in analyzing the cost. (1) Time saving: preliminary results of an observational study indicated that extending the administration interval from three times weekly to once weekly (QW) was associated with substantial time savings [34]. It was estimated that 350 h of physician/nurse time per year could be saved in a center with 50 dialysis patients. If administration intervals could be successfully extended beyond QW for all patients, the resulting time savings could enable health care providers to spend more time focusing on other aspects of CKD management, including patient education, and to address other modifiable risk factors, such as hypertension and mineral balance. (2) The low conversion factor (200:1) with subsequent high doses used as the start of the study could explain in part the total high cost of Darbe; in other words, if we would have used the ratio of (350:1) from the start of the study, we would have saved 175 % of the cost. (3) From the experience of using Darbe and Epo, in cancer patients, taking into consideration the duration of clinical benefit (DCB) that is 2–7 days in Epo and 7–21 days in Darbe, one study showed, after accounting for DCB, that the average weekly cost of darbepoetin alfa was significantly lower than that of epoetin alfa ($619 vs. $940; *p* < 0.001) [35]. There was significant more vascular access thrombosis in the Epo group compared to Darbe group (12 vs. 1.4 %) *p* < 0.001, and vascular access thrombosis was reported similar in Darbe and Epo groups (10 and 9 %, respectively) in a previous study comparing the efficacy and safety of Darbe to Epo [36]. The cause of difference in the thrombosis of vascular access between the two groups in our study is not well clear, and vascular access thrombosis has been reported as a complication in dialysis patient assigned to high Hb target [37]; in our study, the percentage of patients with Hb >125 g/l was similar in both groups (23.9, 23.3 %) and it cannot explain this phenomenon, but one can speculate that the use of decreased doses of Darbe during the study period could have a favorable impact on the access thrombosis. All biological parameters evaluated during the study were comparable between the two groups, and there was no difference between the baseline and end of the study parameters. There was no difference between the two groups in terms of number of hospitalizations and number of deaths.

## Study limitations

1. The two groups were matched for several important confounders; however, other residual confounders, like the presence of inflammation, occult blood loss, missed dose injection, and others, could be there and not matched between the two groups.
2. There was difference in gender between the two groups; yet randomization was done by computer, but patients were not stratified by gender; however, the analysis of subgroups (male and female) did not show any difference between males and females; in addition, we did not find any literature supporting ESAs response difference between males and females.

## Conclusion

Darbepoetin alfa Q weekly or every 2 weeks is more efficient in achieving target Hb, with less dose changes and Hb variability, and much less vascular access complications.

## Appendix: Intravenous iron management protocol

- S. Iron, TIBC, and Ferritin to be checked every 3 months on all patients
- Target: T Sat between 20 and 50 %
- Aim where possible for Serum Ferritin 200–500.

If T Sat <20 % and/or Serum Ferritin <200 give iron loading:

- Venofer 100 mg IV each dialysis for 10 doses
- Repeat iron profile 72 h after completion of iron loading
- Repeat loading if needed or proceed to maintenance dosage Venofer 100 mg IV every 2 weeks.

If T Sat 20–30 % and Ferritin <500

- Start Venofer 100 mg IV weekly × 5 doses and then maintenance of 100 mg IV every 2 weeks.

If T Sat 30–50 %

- Start or continue maintenance dose Venofer at 100 mg IV every 2 weeks
- Continue checking iron profiles every 3-month titrates accordingly.

**Table:**

For T Sat > 50 % or Ferritin > 800	Hold iron (Fe overload)
For Ferritin > 500 and T Sat ≤20 % (inflammation)	Trial of 100 mg Venofer IV weekly × 10 doses and evaluated response

- In case of holding iron, continue checking iron levels every 3 months and treat accordingly

Note:

1. All patients with documented iron deficiency should have stools tested for occult blood.
2. For patients with T Sat <30 % and S. Ferritin >500, suggest inflammation and then trial of iron replacement and evaluate response.

## References

[1] Li S, Foley RN, Collins AJ (2004). Anemia, hospitalization, and mortality in patients receiving peritoneal dialysis in the United States. Kidney Int.

[2] Regidor DL, Kopple JD, Kovesdy CP (2006). et al Associations between changes in hemoglobin and administered erythropoiesis-stimulating agent and survival in hemodialysis patients. J Am Soc Nephrol.

[3] Silverberg D (2003). Outcomes of anaemia management in renal insufficiency and cardiac disease. Nephrol Dial Transp.

[4] Muntner P, He J, Astor BC (2005). Traditional and nontraditional risk factors predict coronary heart disease in chronic kidney disease: results from the atherosclerosis risk in communities study. J Am Soc Nephrol.

[5] Gerson A, Hwang W, Fiorenza J (2004). Anemia and health-related quality of life in adolescents with chronic kidney disease. Am J Kidney Dis.

[6] Perlman RL, Finkelstein FO, Liu L (2005). Quality of life in chronic kidney disease (CKD): a cross-sectional analysis in the Renal Research Institute-CKD study. Am J Kidney Dis.

[7] Xue JL, Ma JZ, Louis TA (2001). Forecast of the number of patients with end-stage renal disease in the United States to the year 2010. J Am Soc Nephrol.

[8] Roderick P, Davies R, Jones C (2004). Simulation model of renal replacement therapy: predicting future demand in England. Nephrol Dial Transp.

[9] Rao M, Pereira BJ (2005). Optimal anemia management reduces cardiovascular morbidity, mortality, and costs in chronic kidney disease. Kidney Int.

[10] KDOQI; National Kidney Foundation (2006). KDOQI clinical practice guidelines and clinical practice recommendations for anaemia in chronic kidney disease. Am J Kidney Dis.

[11] Locatelli F, Aljama P, Barany P (2004). Revised European best practice guidelines for the management of anaemia in patients with chronic renal failure. Nephrol Dial Transp.

[12] Deicher R, Horl WH (2004). Differentiating factors between erythropoiesis-stimulating agents: a guide to selection for anaemia of chronic kidney disease. Drugs.

[13] Macdougall IC, Gray SJ, Elston O (1999). Pharmacokinetics of novel erythropoiesis stimulating protein compared with epoetin alfa in dialysis patients. J Am Soc Nephrol.

[14] Locatelli F, Olivares J, Walker R (2001). Novel erythropoiesis stimulating protein for treatment of anemia in chronic renal insufficiency. Kidney Int.

[15] Elliott S, Chang D, Delorme E, Eris T, Lorenzini T (2004). Structural requirements for additional N-linked carbohydrate on recombinant human erythropoietin. J Biol Chem.

[16] Egrie JC, Dwyer E, Browne JK, Hitz A, Lykos MA (2003). Darbepoetin alfa has a longer circulating half-life and greater in vivo potency than recombinant human erythropoietin. Exp Hematol.

[17] Tsubakihara Y, Hiramatsu M, Lino Y, Akizawa T, Koshikawa S, Group KS (2004) The pharmacokinetics of KRN231 (darbepoetin alfa) after subcutaneous (SC) administration: a comparison between peritoneal dialysis and predialysis chronic renal failure (CRF) patients in Japan. Paper presented at: 41st ERA-EDTA congress, Lisbon, Portugal

[18] Padhi D, Jang G (2005) Pharmacokinetics of Aranesp (darbepoetin alfa) in patients with chronic kidney disease. Paper presented at: 42nd ERA-EDTA congress, Istanbul, Turkey

[19] Padhi D, Ni L, Cooke B, Marino R, Jang G (2006). An extended terminal half-life for darbepoetin alfa: results from a single-dose pharmacokinetic study in patients with chronic kidney disease not receiving dialysis. Clin Pharmacokinet.

[20] Macdougall IC (2002). Optimizing the use of erythropoietic agents—pharmacokinetic and pharmacodynamic considerations. Nephrol Dial Transp.

[21] Locatelli F, Pisoni RL, Akizawa T (2004). Anemia management for hemodialysis patients: kidney disease outcomes quality initiative (K/DOQI) guidelines and dialysis outcomes and practice patterns study (DOPPS) findings. Am J Kidney Dis.

[22] Locatelli F, Aljama P, Barany P (2004). Revised European best practice guidelines for the management of anaemia in patients with chronic renal failure. Nephrol Dial Transp.

[23] Drueke TB, Locatelli F, Clyne N (2006). Normalization of hemoglobin level in patients with chronic kidney disease and anemia. N Engl J Med.

[24] Singh AK, Szczech L, Tang KL (2006). Correction of anemia with epoetin alfa in chronic kidney disease. N Engl J Med.

[25] Locatelli F, Aljama P, Canaud B (2010). Target hemoglobin to aim for with erythropoiesis-stimulating agents: a position statement by ERBP following publication of the trial to reduce cardiovascular events with Aranesp(R) therapy (TREAT) study. Nephrol Dial Transp.

[26] Collins AJ, Brenner RM, Ofman JJ (2005). Epoetin alfa use in patients with ESRD. An analysis of recent US prescribing patterns and hemoglobin outcomes. Am J Kidney Dis.

[27] Fishbane S, Berns JS (2005). Hemoglobin cycling in hemodialysis patients treated with recombinant human erythropoietin. Kidney Int.

[28] Ebben JP, Gibertson DT, Foley RN, Collins AJ (2006). Hemoglobin level variability: associations with comorbidity, intercurrent events, and hospitalizations. Clin J Am Soc Nephrol.

[29] Gibertson DT, Ebben JB, Bradbury B, Dunning SC, Collins AJ (2006). The effect of hemoglobin (Hb) variability and trends on mortality. J Am Soc Nephrol.

[30] Yang W, Israni RK, Brunelli SM, Joffe MM, Fishbane S, Feldman HI (2007). Hemoglobin variability and mortality in ESRD. J Am Soc Nephrol.

[31] Brunkhorst R, Bommer J, Braun J, Haag-Weber M, Gill C, Wagner J, Wagener T (2004). Darbepoetin alfa effectively maintains hemoglobin concentrations at extended dose intervals relative to intravenous or subcutaneous recombinant human erythropoietin in dialysis patients. Nephrol Dial Transp.

[32] Cremieux PY, Audenrode MV, Lefebver P (2006) The relative dosing of epoetin alfa and darbepoetin alfa in chronic kidney disease. Curr Med Res Opin 22(12):2329–2336 10.1185/030079906X154024 17257447

[33] Chan KE, Lafayette RA, Whittemore AS, Hlatky MA, Moran J (2008). Facility factors dominate the ability to achieve target haemoglobin levels in haemodialysis patients. Nephrol Dial Transp.

[34] De Cock E, Van Bellingham L, Standaert B (2002). Assessing provider time for anaemia management of dialysis patients using time & motion methods: a multi-centre observational study in Europe. Value Health.

[35] Song X, Long SR, Marder WD, Sullivan SD, Kallich J (2009). The impact of methodological approach on cost findings in comparison of epoetin alfa, darbepoetin alfa. Ann Pharmacother.

[36] Vanrenterghem Y, Barany P, Mann JF (2002). Randomized trial of darbepoetin alfa for treatment of renal anemia at a reduced dose frequency compared with rHuEPO in dialysis patients. Kidney Int.

[37] Phrommintikul A, Haas SJ, Elsik M, Klum H (2007). Mortality, target haemoglobin concentrations in anemia patients with chronic kidney disease treated with erythropoietin: a meta-analysis. Lancet.

